# Mapping asthma-associated variants in admixed populations

**DOI:** 10.3389/fgene.2015.00292

**Published:** 2015-09-29

**Authors:** Tesfaye B. Mersha

**Affiliations:** Division of Asthma Research, Department of Pediatrics, Cincinnati Children's Hospital Medical Center, University of CincinnatiCincinnati, OH, USA

**Keywords:** admixture mapping (AM), admixed population, genetic ancestry, ancestry-informative markers (AIMs), socio-environmental risk factors, asthma, genome-wide association study (GWAS), next-generation sequencing (NGS)

## Abstract

Admixed populations arise when two or more previously isolated populations interbreed. Mapping asthma susceptibility loci in an admixed population using admixture mapping (AM) involves screening the genome of individuals of mixed ancestry for chromosomal regions that have a higher frequency of alleles from a parental population with higher asthma risk as compared with parental population with lower asthma risk. AM takes advantage of the admixture created in populations of mixed ancestry to identify genomic regions where an association exists between genetic ancestry and asthma (in contrast to between the genotype of the marker and asthma). The theory behind AM is that chromosomal segments of affected individuals contain a significantly higher-than-average proportion of alleles from the high-risk parental population and thus are more likely to harbor disease–associated loci. Criteria to evaluate the applicability of AM as a gene mapping approach include: (1) the prevalence of the disease differences in ancestral populations from which the admixed population was formed; (2) a measurable difference in disease-causing alleles between the parental populations; (3) reduced linkage disequilibrium (LD) between unlinked loci across chromosomes and strong LD between neighboring loci; (4) a set of markers with noticeable allele-frequency differences between parental populations that contributes to the admixed population (single nucleotide polymorphisms (SNPs) are the markers of choice because they are abundant, stable, relatively cheap to genotype, and informative with regard to the LD structure of chromosomal segments); and (5) there is an understanding of the extent of segmental chromosomal admixtures and their interactions with environmental factors. Although genome-wide association studies have contributed greatly to our understanding of the genetic components of asthma, the large and increasing degree of admixture in populations across the world create many challenges for further efforts to map disease-causing genes. This review, summarizes the historical context of admixed populations and AM, and considers current opportunities to use AM to map asthma genes. In addition, we provide an overview of the potential limitations and future directions of AM in biomedical research, including joint admixture and association mapping for asthma and asthma-related disorders.

## Introduction

### Asthma: its importance, prevalence, and racial disparities

**Figure 1:**
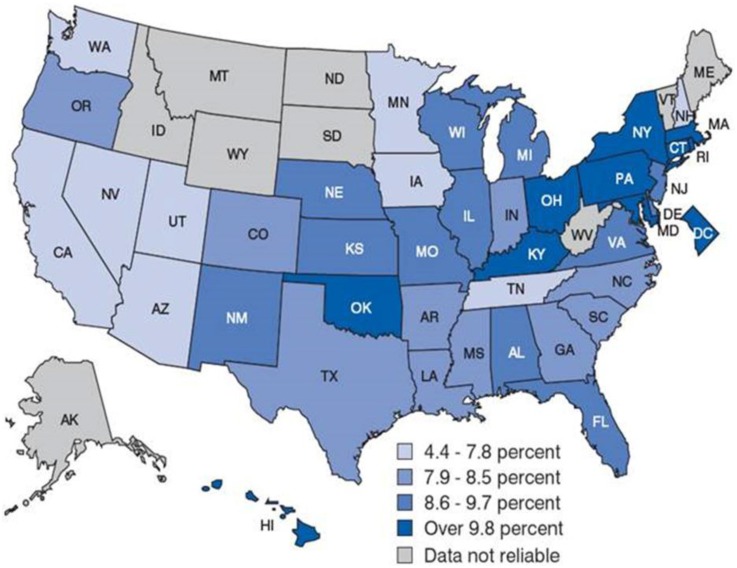
**Childhood asthma prevalence by state-by-state in the United States**. Asthma prevalence rates are generally higher in the Northeast region. This could attribute to the population composition. For example, the Puerto Rican population, in which asthma prevalence is highest, tends to be concentrated in the Northeast region of the country. Source: CDC/NCHS, National Health Interview Survey, annual average for the period 2001–2005.

Asthma is the most common chronic illness affecting children in the United States (CDC, 2012). It is a major public health problem that affects up to 315 million individuals worldwide and 40 million people in the United States, including 11 million children (Akinbami et al., 2012; WHO, 2013). Asthma is the 3rd-ranking cause of hospitalization among children less than 18 years old, and it is a leading cause of school absences. According to the Centers for Disease Control and Prevention (CDC, 2012), this translates into more than 14 million office visits, 2 million emergency department visits, 500,000 hospitalizations, and more than 3300 deaths each year. On average, children and adult missed 10 and 14 million days of school and work due to asthma, respectively. The cost of asthma to the health care system surpasses $56 billion per year (Sculpher and Price, 2003; Akinbami et al., 2011; Barnett and Nurmagambetov, 2011; CDC, 2012). Figure 1 shows the state-by-state prevalence of asthma across the United States. Asthma prevalence rates are generally higher in the Northeast region, which has a population with a higher degree of African ancestry as compared with European ancestry (Bryc et al., 2015). Although disparities in asthma rate clearly reflect complex interactions among socioeconomic factors (e.g., income, education, insurance) as well as physical and environmental exposures (e.g., traffic, cigarette smoke), the contribution of genetic ancestry factors is substantial. Heritability estimates of between 36 and 79% support the genetic contribution to asthma, yet relatively little is known about causal variants, racial variation, and the pathways that contribute to asthma etiology (Akinbami et al., 2011). Twin and family studies showed that asthma runs in families. Although positive family history is the strongest risk factor for asthma, the transmission of asthma from parents to offspring does not follow simple Mendelian inheritance. Rather, a polygenic, multifactorial inheritance, which underscores the genetic and non-genetic basis of asthma, both of which are incompletely understood (Mathias, 2014).

**Figure 2:**
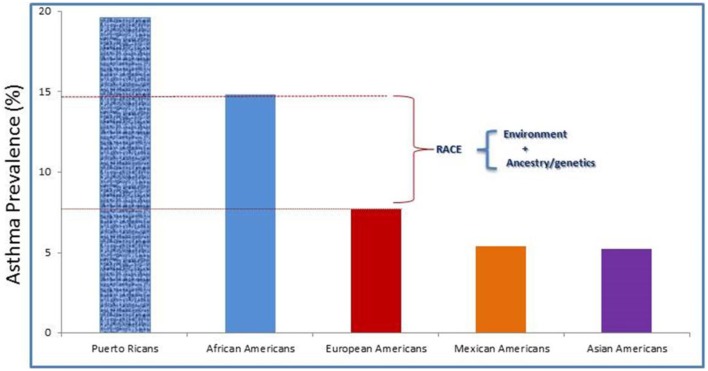
**Asthma prevalence rates by race/ethnicity among the U.S. children**. Race is considered as risk factor for asthma. But, how much is due to environmental exposures, genetic, and asthma morbidity factors required further studies.

Racial disparities exist with regard to prevalence and drug response in asthma. Its prevalence in Puerto Ricans (19.6%) and African Americans (14.6%) is higher than among European Americans (8.2%), Mexican Americans (4.8%), and Asian Americans (4.2%) (Gupta et al., 2006; Moorman et al., 2007; Baye et al., 2011b; Silvers and Lang, 2012) (http://cvp.ucsf.edu/docs/asthma_factsheet.pdf) (Figure 2). Although Mexican American children have a low prevalence of asthma (4.8%) and better lung function, they—in addition to Puerto Ricans and African Americans—experience an excess of asthma-related symptoms, missed school days, and unplanned health care visits. African Americans, for instance, are four times more likely to be hospitalized and seven times more likely to die from asthma than their European American counterparts (Akinbami et al., 2009, 2014). Federal efforts to reduce these disparities include Healthy People 2020 and a coalition of federal agencies formed under the auspices of the President's Task Force on Environmental Health Risks and Safety Risks to Children (www.epa.gov/childrenstaskforce). Variations in asthma prevalence among subpopulations suggest that asthma is a heterogeneous disease with varied risk profiles (Miller, 2000). These population differences can have clinical implications; an example of this is the use of long-acting β2-agonists for patients with asthma. Although combining corticosteroid treatment with the use of a long-acting β2-agonist can lead to better asthma control for most patients, a small proportion of patients—including African-Americans—appear to be at increased risk for adverse and even fatal outcomes from the use of long-acting β2-agonists (Khianey and Oppenheimer, 2011). The reason for this is still unknown. However, some studies have suggested that β2-adrenergic receptors genotype—ancestry as well as environment interactions may be responsible (Elbahlawan et al., 2006; Blake et al., 2013). Admixed population is an idea population to investigate the contribution of genetic ancestry and environmental exposures with regard to modifying asthma risk.

### Admixture and admixed populations

The term *admixture* refers to the process in which individuals from two or more geographically isolated populations with different allele frequencies mate and form a new mixed or “hybrid” population (Reed, 1969; Chakraborty, 1986). It is the result of gene flow over time between genetically distinct human populations that leads to a mosaic of chromosomal segments that represent each population. The world is becoming highly multiethnic, and intermarriage between different groups is becoming more and more common (Hellenthal et al., 2014; Mersha and Abebe, 2015). As such, admixed populations exist in several regions of the world today (Hellenthal et al., 2014; Bryc et al., 2015). In the United States in particular, there are two large admixed populations: African Americans and Latino Americans. Populations like African Americans, and Latino Americans were formed in the past 400 years (Smith et al., 2004). African Americans were derived from an admixture event dating back to the 16th century and the trans-Atlantic slave trade. Today, on average, African Americans have genomic segments of ~80% African ancestry and ~20% European ancestry (Patterson et al., 2004; Reiner et al., 2005). Latino American populations are admixed and are the result of three-way admixture events between European, African, and Native American ancestries (Collins-Schramm et al., 2002; Bonilla et al., 2004). With regard to admixture history, the Latino populations are much more diverse as compared with African Americans, and their proportions of admixture vary significantly by geographical region (Collins-Schramm et al., 2002; Bonilla et al., 2004; Parra, 2006; Bryc et al., 2015). For example, Mexican Americans generally have a higher genomic segment of Native American ancestry (ranging between 35 and 64%) but lower genomic segments of African ancestry (ranging from 3 to 5%) as compared with Puerto Ricans, whose Native American ancestry ranges between 12 and 15% and whose African ancestry ranges between 18 and 25% (Brehm et al., 2012). The prevalence of asthma is highest among Puerto Ricans and lowest among Mexican Americans which could be due to higher proportion of African ancestry among Puerto Ricans than Mexican Americans (see Figure 2); this phenomenon is referred to as the “Latino paradox” (Cagney et al., 2007). In an admixed population, the effects of individual loci on asthma can be detected by (1) using admixture mapping—between local ancestry and phenotype for ancestry-specific variants; (2) using association analysis—between genotype and phenotype for ancestry-shared variants. Admixture mapping-based approaches is more powerful for cases in which the causative loci exhibit large allele frequency differences in ancestral populations, whereas genotype-based association approaches are expected to be more powerful for cases in which the causative loci have similar allele frequencies in ancestral populations.

### Admixture mapping in admixed populations

**Figure 3:**
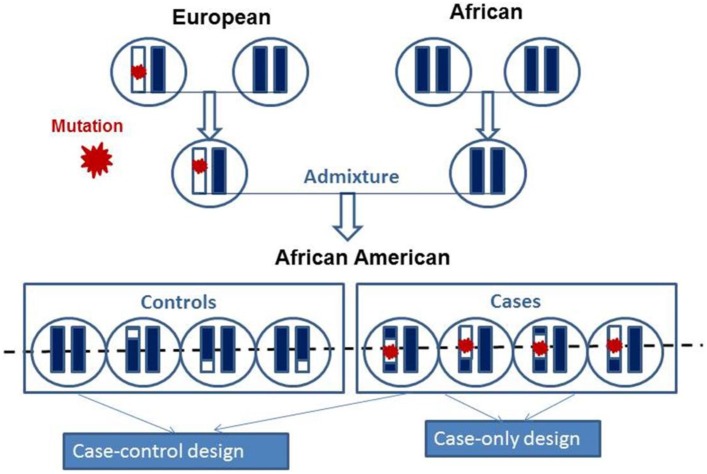
**Schematic presentations of the mosaic chromosomal structures of admixed population derived from two founders**. The chromosomes of the two founders are combined and after several generations of random mating produce present day admixed individual. Admixed population can be studied using case-control and case-only admixture mapping study design to map mutation as indicated by red star.

Admixture mapping (AM) is a gene mapping method used for persons of mixed ancestry to identify genomic segments in which the proportion of a particular ancestry is strikingly higher or lower than that seen elsewhere in their genomes (Patterson et al., 2004; Smith and O'Brien, 2005). Such enriched genomic regions from a given ancestry among cases would indicate the presence of ancestry-related genetic risk variants (Figure 3). At a specific site in the genome, if two ancestral populations have differences in disease risk allele (e.g., high risk in Europeans and low risk in Africans), then an African American in the admixed population with more European ancestry tends to have a higher risk than a person in the same population with less European ancestry. Thus, if an ancestral population carries a genetic risk allele at a higher frequency, then the genomes of affected offspring in this ancestral population will share a greater level of ancestry at such disease susceptibility locus compared with the background ancestry (i.e., genome-wide average) (Darvasi and Shifman, 2005). The underlying argument of admixture-based gene mapping is that, when two or more populations with different genetic architecture interbreed, long segments of DNA (haplotypes) that have distinguishable ancestral origins will be created. Importantly, marker coverage and sample size required for AM depends on how many generations passed since admixture. The larger the genomic segments of particular ancestry, the less marker saturation and the smaller the sample sizes required (as compared with standard association studies) to localize the genes that contribute to asthma (Hirschhorn and Daly, 2005; Smith and O'Brien, 2005). Shriner et al. (2011) reported a reduction in sample size of 64% to reach genome-wide significance with AM as compared with association mapping.

Admixture-based gene mapping in humans is similar to the crossing of plants and animals. In both cases, the starting point involves two (or more) genetically separated populations that have been intermixed through several generations of mating to form an admixed population. The genetic structure of the new population is a mixture of the genetic materials of the founding populations. In the experimental genetics of inbred lines, phenotypically distinct strains are used. Using AM to correlate phenotypes to the originating populations, differentially selected traits may be identified in the two founding populations. There are important differences between mapping in an admixed population and mapping with crosses of inbred strains. Whereas, inbred strain genetic mapping is mainly experimental, human genetic mapping is observational (i.e., there may be no complete information about the length of the generations being studied).

### Why might admixture mapping work for asthma in admixed populations?

The concept of using admixed populations to localize disease-associated genes was proposed more than 50 years ago (Rife, 1954), but it gains momentum recently due to the accessibility of genome-wide dense markers including highly ancestry-informative markers (AIMs) and adequate statistical tools (International Hapmap, 2005; McKeigue, 2005; Smith and O'Brien, 2005). The arguments in favor of AM are compelling, and the statistical methods are improving rapidly (McCarty et al., 2005, 2008; McKeigue, 2005). The theme in favor of AM is compelling and best applied when the prevalence of a disease is significantly different between the ancestral populations. In such situation, individuals who are carrying the disease are expected to show an elevated genomic contribution around the disease loci within the admixed genome. The African American admixed population structure presents unique opportunities to explore the genetic etiology of complex diseases such as asthma because of the recent mixture between African and European populations (on average, 10 generations ago) and to look at the variation in asthma prevalence between African and European ancestry (Patterson et al., 2004). Despite advances in asthma care, African Americans are four times more likely to be hospitalized and seven times more likely to die from asthma than European Americans (Akinbami et al., 2009, 2014). The study of admixed populations allows for analysis that is not possible within homogeneous groups. This includes the analysis of single nucleotide polymorphisms (SNPs), which do not differ within the ancestral populations but which have different allele frequency in different ancestral populations and which require an admixed sample to detect their relationship with the phenotype.

Several variants associated with complex traits in admixed populations have been identified with the application of AM. These include risk variants associated with cardiovascular disease (Zhu et al., 2005; Zhang et al., 2008b); multiple sclerosis (Reich et al., 2005); prostate cancer (Freedman et al., 2006); serum IL-6 levels (Reich et al., 2007); and asthma in populations of African, Latino, Mexican, and Puerto Rican ancestry (Salari et al., 2005; Choudhry et al., 2008; Mathias et al., 2010; Torgerson et al., 2011, 2012; Drake et al., 2014; Galanter et al., 2014; Pino-Yanes et al., 2014). These results indicate that the admixed population provides an excellent opportunity to harness the power of linkage disequilibrium (LD) represented by ancestral markers transmitted together, thereby making use of disease prevalence in the ancestral (founder) populations. Although the exact numbers of AM studies so far are difficult to establish, several reported studies involved Latino populations compared with African American populations (Table 1).

**Table 1:** **Admixture peaks containing ancestry-specific asthma susceptible genes identified by admixture mapping (AM)**.

**Population**	**Genomic region**	**Ancestry**	**Associated gene(s)**	**Reference(s)**
African American	1q23.1	African	PYHIN1	Torgerson et al., 2011
African American	5q33	African	ADRA1B	Mathias et al., 2010
African American	2q12.3–q14.2	African	DPP10	Mathias et al., 2010
African American	20p12	African	PRNP	Mathias et al., 2010
Mexican	2q14.1	European	DPP10	Torgerson et al., 2012
Mexican	4q22.1	African	FAM13A	Torgerson et al., 2012
Mexican	5q32–q33.1	Native American	SPINK5,SCGB3A2	Torgerson et al., 2012
Mexican	1p13	N/A	SLC22A15	Drake et al., 2014
Latino	6q15	African	N/A	Torgerson et al., 2012
Latino	8q12	African	N/A	Torgerson et al., 2012
Latino	6p21	Native American	MUC22, PSORS1C1	Galanter et al., 2014
Latino	17q21	European	IKZF3, IL1RL1, TSLP, IL33, ORMDL3	Torgerson et al., 2011
Latino	6p21.32–p22.1	Native American	HLA-B	Pino-Yanes et al., 2014
Latino	13p22–31	African	N/A	Pino-Yanes et al., 2014
Latino	14q23.2	African	GPHB5	Pino-Yanes et al., 2014
Latino	22q13.1	African	N/A	Pino-Yanes et al., 2014
Puerto Rican	5q23.3	African	N/A	Choudhry et al., 2008
Puerto Rican	13q13.3	African	N/A	Choudhry et al., 2008
Puerto Rican	7q11.2	Native American	EGFR	Torgerson et al., 2012
Puerto Rican	7q31.3–31.31	Native American	CFTR	Torgerson et al., 2012
Puerto Rican	4q13.1	African	MUC7	Torgerson et al., 2012
Puerto Rican	5q31.2	Native American	EGR1	Torgerson et al., 2012
Puerto Rican	5q33.3	Native American	IL12B	Torgerson et al., 2012

## Procedures for conducting admixture mapping analysis

AM using admixed population involves four main steps: (1) select a panel of AIMs or high-density SNPs that differentiate well between the ancestral populations; (2) ancestry estimation. Genotype asthmatic and control subjects using the selected AIMs or a high-density SNPs panel followed by inferring admixture proportions; (3) admixture mapping using cases-only or cases and controls analysis. Ancestral estimates will be used to search for an aberration toward the ancestral population with a higher disease risk locus using the AM procedure; and (4) the prioritization of variants in AM peak region using step-wise regression and conditional analyses.

### Step 1: selection of a panel of ancestry-informative markers or high-density single nucleotide polymorphisms

The idea that genetic markers are present at different allele frequencies in different ancestral populations was reported over 40 years ago (Reed, 1973). Neel (1974) referred to these markers as “private.” Reed (1973) used the term *ideal* (in reference to their utility for individual ancestry estimation) to describe the alleles fixed in different populations. Chakraborty et al. (1991) called those variants “unique alleles,” and they demonstrated how frequencies of such “unique” alleles in an admixed population could be used to provide a likelihood estimate of the “hereditary” proportion of disease prevalence differences in populations. Markers with different frequency distributions among ancestral populations are considered as AIMs, and they are used to map ancestry-related genes in admixed populations because they can distinguish the ancestral origin of the haplotype on which they reside. Informativeness is the amount of information that is imparted by such markers (Shriver et al., 2003). The distinctive distribution of these alleles results from the history of human population migration. As human populations grew and fanned out over the globe from Africa several hundred thousand years ago, populations dispersed and settled farther and farther away from one another (Bamshad et al., 2004). During this time period, genetic changes occurred, including spontaneous mutations that could be passed down from generation to generation in each population. Depending on how close populations lived to one another, they may share few or many of these alleles.

AIMs are used to estimate the geographical origins of an individual's ancestors, which is typically expressed as the proportion of ancestry from different continental regions (Ding et al., 2011b). Any marker differentiating racial ancestry can be used for ancestry estimation and for AM. Although autosomal SNPs are commonly used as genetic markers to infer ancestry, polymorphic sites of mitochondria, Y-linked DNA markers, and X-linked markers are also important for providing separate stories of the ancestry of individuals from paternal and maternal lineages (Rohl et al., 2001; Phillips et al., 2007; Mersha and Abebe, 2015). SNPs have become the markers of choice for locating disease genes: they are abundant and stable, they are easier than other existing markers to genotype, and they are informative for LD when selected for appropriate allele frequencies. The ideal AIM should have alleles that are fixed between the two ancestral populations and that thus have fixation index (F_ST_) value of 1.0 (Ding et al., 2011a). Measures that precisely quantify the amount of information that each locus contributes to the inference of ancestry or population structure are highly relevant for the selection of informative markers (Pritchard and Donnelly, 2001). These methods lead to a great reduction in genotyping required for ancestry inference. Over the years, several measures of marker informativeness (i.e., the ability of markers to differentiate between ancestral populations) have been developed to select the most informative AIMs from an ever-increasing wealth of genomic databases (Shah and Kusiak, 2004; Baye et al., 2009). Ding et al. (2011b) compared the performance of measures of marker informativeness, including Fisher information content, Shannon information content, F statistics, informativeness for assignment measure, and the absolute allele frequency differences. In addition, Amirisetty et al. (2012) developed *AncestrySNPminer*, the first Web-based bioinformatic tool designed to retrieve AIMs from public databases (e.g., HapMap, 1000 Genomes Project).

While sparse ancestry informative markers (AIMs) are more cost effective and can estimate global ancestry proportion, high-density SNP markers are required for local ancestry estimation. The high-density genome-wide markers provide an increased sensitivity to smaller ancestry segments and higher resolution of ancestry switches (i.e., changes in ancestry in the interval between two markers) than a sparse panel of AIMs (Shriner et al., 2011; Jin et al., 2014). By assessing local ancestry for every variant (taking LD patterns into account) from high-density SNP chips rather than relying on local ancestry estimates from a small number of AIMs, researchers have the opportunity to prioritize and evaluate potential variants directly in each admixture peak.

### Multipoint ancestry-blocks analysis improve power

Although human genetic variations reflect differences at single alleles as well as at the haplotype level, most ancestry estimators use allele frequency (locus-by-locus) data between parental contributions along the chromosome and ignore the molecular information that is available in the ancestry-haplotype block structures in the genome. Individual mutations carry only weak signals as they apply to population ancestry. Inferring information about admixture proportions by combining information from across multiple loci that form haplotypes or haploblocks is quite valuable. By adding information across the whole genome at the haplotype level, these admixture events can be reconstructed more accurately (Jobling et al., 2004). However, previous methods have not taken into account multiple loci as provided by haplotype structures in ancestral populations. Multipoint ancestral haplotype advantages include their use of more information from the data when a susceptibility variant in the region is untyped or partially typed, improve comparability of results across studies for localization. As a result, multipoint ancestral haplotype block methods have the potential to vastly improve power as compared with single-point methods (Giardina et al., 2008).

### Step 2: ancestry estimation

Ancestry can be viewed at populations, at individuals within a population, and at a locus within individual levels. In gene mapping, the goal of ancestry estimation is to determine individual's ancestral origin at the global and local (chromosome segment) levels. Theoretically, it is possible to measure every point in the genome and determine ancestry. However, recombination events at each intermixing stage cannot be observed directly (Sankararaman et al., 2008; O'Reilly and Balding, 2011), and complex pedigrees and founder information (the number of ancestral or founders) is usually unknown. Thus, it is of interest to estimate the ancestry of resulting DNA sequences at each position.

#### Local and global ancestry

Chromosomes of an individual with admixed ancestry represent a mosaic of chromosomal blocks from the ancestral populations, and they can be inferred at the global and local levels using AIMs or high-density SNPs. The ancestral origin at a particular locus is referred as *local ancestry.* Local ancestry is concerned with the locus-by-locus ancestry of a genomic segment given reference population data (Pasaniuc et al., 2009; Price et al., 2009). Local ancestry might provide better coverage of rare variation because rare variants are more likely to differ in frequency between populations with varying demographic histories (Gravel et al., 2011). *Global ancestry* involves estimating the proportion of local ancestry averaged across the entire genome (oraverage proportion of each contributing population across the genome (Falush et al., 2003; Patterson et al., 2006; Alexander et al., 2009). Global ancestry is often used as a covariate to correct for population stratification in genetic analysis, because it roughly reflects differences in allele frequencies between continental populations.

### Tools to estimate local and global ancestry

Local ancestry methods, at each position in the genome, estimate how many copies (0, 1, or 2) were inherited from prespecified ancestral populations (see Figure 3). Simply put, they identify which parts of the DNA sequence were inherited from each ancestral population. Local ancestry mapping focuses on particular segments of a genome and determines from which ancestral lineage these segments were most likely inherited. The hidden Markov model–based algorithm is used traverse each marker and attempt to assign an ancestral state to each chromosomal block by considering the allele frequencies of the marker and the surrounding markers, which are incorporated as “transition probabilities” from each previous SNP. Through a series of probabilistic computations, these predicted transmission probabilities generate the most likely ancestry for a haplotype. Several methods have been proposed to estimate local and global genetic ancestry in admixed individuals using AIMs (or sparse markers) on reference allele frequencies for each parental population [e.g., Local Ancestry in adMixed Populations (LAMP)] (Sankararaman et al., 2008); on reference haplotypes for each of the ancestral populations from high-density SNPs such as HAPMIX (Price et al., 2009) and LAMP-LD (Baran et al., 2012); or sequence data such as SEQMIX (Hu et al., 2013) and NGSadmix (Hu et al., 2013).

Global ancestry is estimated as the proportion (percentage) of ancestral blocks from each contributing population across the markers of interest. Several tools are used to infer global ancestry. LAMP-LD uses high-density SNP data and incorporates LD information when estimating local ancestry from two or more ancestral populations (Baran et al., 2012). LAMP-LD performs better than LAMP (Chen et al., 2014; Yorgov et al., 2014), which relies on set of AIMs that are in low LD (Sankararaman et al., 2008). HAPMIX uses haplotype information to infer local ancestry in admixed samples (Price et al., 2009). STRUCTURE, perhaps the most widely used program for estimating global genetic ancestry, was developed by Pritchard et al. (2000a). It is a model-based clustering approach which utilizes genotype data to identify admixture proportions at the individual level. A maximum likelihood estimation method such as ADMIXTURE can also be used to estimate global ancestry (Alexander and Lange, 2011). Other methods, such as EIGENSTRAT, compute principal components by comparing African American genotypes with 1000 Genomes Project reference populations YRI and CEU and by correcting for global ancestry variations between continental populations (Price et al., 2006). Summaries of admixture estimations (or control values for population stratification) and AM software packages are shown in Table 2.

**Table 2:** **Lists of publicly available software usefulness in developing ancestry informative markers, global and local ancestry inferences, and admixture mapping with a link to software website**.

**Software**	**Link**
ADMIXTURE	http://www.genetics.ucla.edu/software/admixture/index.html
ADMIXMAP	http://www.ucd.ie/genepi/software.html
ALDER	http://groups.csail.mit.edu/cb/alder/
ALDsuite	https://github.com/johnsonra/ALDsuite
ANCESTRYMAP	http://genepath.med.harvard.edu/~reich/Software.htm
ANCESTRYSNPMINER	https://research.cchmc.org/mershalab/AncestrySNPminer/login.php
DBM-Admix	http://sites.stat.psu.edu/~yuzhang/software/
EIGENSTRAT/smartpca	http://www.hsph.harvard.edu/faculty/alkes-price/software/
EILA	http://cran.r-project.org/
ELAI	https://www.bcm.edu/research/labs/statistical-genetics-lab/software
FRAPPE	http://med.stanford.edu/tanglab/software/frappe.html
GEMTools	http://www.wpic.pitt.edu/wpiccompgen/GemTools/GemTools.htm
HAPAA	http://hapaa.stanford.edu
HAPMIX	http://www.stats.ox.ac.uk/~myers/software.html
iAdmix	https://sites.google.com/site/vibansal/software/iAdmix
LAMP	http://lamp.icsi.berkeley.edu/lamp/
LAMP-LD	http://lamp.icsi.berkeley.edu/lamp/lampld/
LASER	http://csg.sph.umich.edu/chaolong/LASER/
parLEA	http://dm.unife.it/parlea
MaCH-admix	http://www.unc.edu/ yunmli/MaCH-Admix/
MEADMIX	http://www.mybiosoftware.com/meadmix-1-0-molecular-estimator-admixture.html
MULTIMIX	http://mathgen.stats.ox.ac.uk/genetics_software/multimix/multimix.html
NGSadmix	http://www.popgen.dk/software/index.php/NgsAdmix
PCAdmix	https://sites.google.com/site/pcadmix/home
PSMIX	http://zhaocenter.org/labcode/PSMix/psmixreadme.txt
RFMix	http://med.stanford.edu/bustamantelab/
SABER	http://med.stanford.edu/tanglab/software/saber.html
SEQMIX	http://genome.sph.umich.edu/wiki/SEQMIX
SNAP	https://www.broadinstitute.org/mpg/snap/ldsearchpw.php
SPSmart	http://spsmart.cesga.es/hapmap.php?dataSet=hapmap
STRUCTURE	http://pritchardlab.stanford.edu/software.html

### Step 3: admixture mapping: case-only or case-control analysis

AM is designed to detect genomic signals by correlating disease prevalence with the admixture proportions estimated by AIMs or high-density SNPs (Chakraborty and Weiss, 1988). Estimation of the local ancestry of admixed population at every locus or individual provides the basis for AM. In case-control design, the ancestry is compared at a given locus between the cases and the controls (McKeigue, 1998). In case-only design, local ancestry estimates of the cases at a given locus is compared with the global ancestry within the cases (Figure 3) (Montana and Pritchard, 2004; Mexal et al., 2010; Zhu, 2012). Since there is no statistical noise introduced by the controls, the case-only approach is more powerful than the case-control approach. However, its power requires that no null loci exist with deviations due to selection since admixture (Seldin et al., 2011).

### Test statistics for case-only and case-control admixture design

When mapping asthma genes using AM, the primary test will be the association of asthma with local ancestry at a locus. Zhu (2012) outlined the test statistics for the case only design: $Z_{C}\left(t\right)=\left({\hat{\pi }}_{d}\left(t\right)-{\hat{\pi }}_{d}\left(\theta =0.5\right)\right)\;∕\;\left(\sigma \left({\hat{\pi }}_{d}\left(t\right)\right)\right)$, and for the case-control design: $Z_{CC}\left(t\right)=\left[\left({\hat{\pi }}_{d}\left(t\right)-{\hat{\pi }}_{d}\left(\theta =0.5\right)\right)-\left({\hat{\pi }}_{c}\left(t\right)-{\hat{\pi }}_{c}\left(\theta =0.5\right)\right)\right]\;∕\;\left(\sigma \left({\hat{\pi }}_{d}\left(t\right)-{\hat{\pi }}_{c}\left(t\right)\right)\right)$, where π_d_(θ) and π_c_(θ) be the proportions of alleles that are from ancestral population X among cases and controls in the current admixed population (*c*), respectively, *t* represent the chromosome location, and θ represents the genetic distance between the disease location and the candidate marker. The null hypothesis is θ = 0.5 between a marker locus and a disease locus (the marker is unlinked to the disease risk). In both models, regions with statistically significant regression coefficients for local ancestry are inferred to harbor disease modifying genes. The case-only or case-control admixture tests are not affected by population structure, because the excess of ancestry is being tested at a marker position (Montana and Pritchard, 2004).

In both case-only and case-control models, targeted regions with statistically significant regression coefficients for local ancestry are inferred to harbor disease associated loci. A target admixture signal region in AM is defined as a 1-unit drop region from a peak of –log10 (*P*-value) (Zhu, 2012). Estimates of local ancestry may be highly correlated. Case-only or case-control admixture tests are not affected by population structure, as excess of ancestry is being tested at a marker position (Montana and Pritchard, 2004). To determine statistical significance, the numbers of independent tests in all of the regions under consideration have to be considered. For instance, on a given chromosome a block of ancestry from one ancestral population can be up to several mega bases long. Thus, the total number of tests is much less than the number of tests in GWASs (which generally requires larger number of participants). This is an important advantage of AM, which reduces the penalty as a result of a larger number of multiple comparisons.

Although appropriate genome-wide significance for AM remains data dependent, local ancestry estimates can be highly correlated and independent blocks of local ancestry must be considered rather than simply the total number of SNPs (Sha et al., 2006). Shriner et al. (2011) estimated the effective number of independent tests based on fitting an autoregressive model to the local ancestry data and evaluating the spectral density at frequency zero. A Bonferroni correction was applied to calculate an adjusted significance threshold to yield an experiment-wise type I error rate of 5%. A conservative estimate of genome-wide significance for local ancestry was used to be 1.2 × 10^−6^ (0.05 divided by 38,566, which is the number of admixture tests) (Parker et al., 2014). On the basis of previous simulation results, a nominal *P*-value 7 × 10^−6^ yielded a genomewide type I error of 0.05 (Tang et al., 2010). Others conducted follow-up admixture peak region with a nominal *p*-value < 1 × 10^−3^ (Gomez et al., 2015).

### Tools for admixture mapping

Well-established first generation admixture analysis tools for from sparse AIMs panels (e.g., ANCESTRYMAP, ADMIXMAP) are used in asthma to identify genomic regions that differ significantly between ancestries (Hoggart et al., 2004; Patterson et al., 2004). Like local and global ancestry tools, Hidden Markov Models estimate individual, population and locus level admixture and test for a relationship between disease risk and individual (or locus) level admixture in case-control and case-only studies. An underlying assumption is the absence of linked, i.e., low LD between markers (Wise et al., 2012). ANCESTRYMAP compares no ancestry effect with an ancestry effect on asthma risk (Patterson et al., 2004). Parameters of 1000 for burn-in iterations and 2000 for follow-on iterations are recommended for Markov chain Monte Carlo runs (Patterson et al., 2004). A log-genome score of more than 1.0 is considered suggestive evidence for the association, whereas a score of more than 2.0 is considered to represents genome-wide significance. Similarly, ADMIXMAP use 1000 burn-in iterations and 4000 follow-on iterations (Hoggart et al., 2004). ADMIXMAP implements a score test that compares ancestry in each chromosomal position with genome-wide ancestry. *Z* statistics is used for significance test, with |*Z*| > 3.0 (suggestive) and |*Z*| > 4.0 (significant) (Hoggart et al., 2004). Once a disease locus is identified based on these tests, a fine mapping analysis is needed to identify specific variants most strongly associated with the disease outcome (Wise et al., 2012). These programs were developed for traditional AM on AIM panels, and not well evaluated for local genetic ancestry estimates using dense panels of markers at genome scale. Ancestry software which gives an accurate estimation of local ancestry at high density markers level are critically important as we move to next generation sequencing studies in admixed populations. Recently, developed tools for high density marker data include HAPMIX (Price et al., 2009) and LAMP-LD (Baran et al., 2012); MULTIMIX (Churchhouse and Marchini, 2013) for genotype/haplotype data, or SEQMIX (Hu et al., 2013) and NGSadmix (Hu et al., 2013) for sequence data. However, further evaluation is required including computational efficiency for next generation whole genome sequencing datasets. A recent R based ALDsuite has functionality that account for local LD using principal components of haplotypes from ancestral population data as well as quality control and downstream analysis of results and visualization graphics (Johnson et al., 2015).

### Step 4: prioritization of variants in admixture mapping peaks using conditional analysis

AM estimates ancestry at genetic markers, which represent the surrounding genomic region; therefore AM does not have the resolution of a GWAS. Hence, once a disease associated admixture signal is identified from previous step, a fine mapping analysis is required to identify specific variants most strongly associated with asthma. To fine-map these AM regions in an effort to reveal loci or variants that contribute to population-level asthma differences, conditional single SNP association analysis can be performed for all loci in each region. Two criteria are used to evaluate loci that “explain” an AM peak region (Shriner et al., 2011). (1) Loci should show suggestive association with the trait conditioning on the genome-wide ancestry (global); (2) these loci should substantially reduce the local ancestry–asthma association. To test the first criterion, variants must have genome-wide significance of *P* < 10^−6^. To assess the second criterion, a joint regression model that includes both local ancestry and SNP genotype in addition to all covariates adjustment is used, and the *P*-value for local ancestry must be less significant as compared with that of the model without the SNP genotype. For regions in which multiple variants or genes meet both criteria, stepwise regression is performed to prioritize a set of variants that may jointly explain the local ancestry association. To search for any additional variants that contribute to these effects, one can further perform conditional analyses while adding the most significant variant as a covariate in the regression model. This analysis is finalized by ranking *P*-values and identifying the most significant associations between local ancestry and disease traits (Patterson et al., 2004). After association is established, association mapping approaches are applied for finer-level resolution, which helps to hone in on the particular variant underlying the association and to validate the original AM result.

In a fine mapping analysis both ancestral and genotype data from admixed population are included in the model, and an association between genotype and disease is the primary test being investigated. Link (Y) ~ β_0_ + β_1_(genotypes) +β_2_(local ancestry) +β_3_(global ancestry) +β_4_(covariates). The generalized linear models are flexible, allowing for multiple types phenotypes (e.g., continuous, dichotomous) and covariates to be included (Johnson et al., 2015).

### Genome-wide associations in admixed populations

Most GWASs have been conducted in individuals of homogeneous populations (e.g., those of European descent) (Ding et al., 2013). European-derived populations are good candidates for GWASs as a result of the relative homogeneity of their genetic origins. GWASs involve scanning thousands of case-control cohorts, which use hundreds of thousands of SNP markers in the human genome. Algorithms are then applied to compare the frequencies of SNPs or haplotype markers between the disease and the control cohorts. Collecting clinical phenotypes from matching cases and control groups as reflected by geographic origin and ethnicity is of critical importance to the success of GWASs. However, the world is continually becoming highly admixed, and allele frequencies are known to vary widely within and between populations; these differences are widespread throughout the genome (International Hapmap, 2005). Thus, it is becoming difficult to find recruits for traditional GWASs because of the highly admixed nature of world populations. GWASs of admixed populations offer the promise of discovering genetic variations that would be missed by the exclusive study of European populations. However, in GWASs of admixed populations, the heterogeneity of the genetic background can lead to spurious associations; this is also referred to as *genetic confounding* caused by population stratification (Price et al., 2006; Baye, 2011; Baye et al., 2011c). Statistical methods (e.g., local and global ancestry inferences) have been employed to overcome or make use of such ancestral variations to identify disease-associating loci in diverse populations. Accordingly, GWASs in African and Latino Americans and other admixed populations are now underway. Although admixed populations require a more careful approach, considering the origin of the chromosomal regions, the vast genetic diversity and population history may be better viewed as an opportunity for new insights. For example, the degree of LD is less in individuals of African descent because of the many generations of recombination in this population (Tishkoff and Kidd, 2004; Bedoya et al., 2006; Price et al., 2007; Tishkoff et al., 2009). For GWASs of populations of European descent, associated SNPs are more often considered as a proxy for the functional variants. For individuals with a lesser degree of LD (e.g., many of those of African descent) the SNPs that are associated with the disease are more likely to be localized much more closely to the biologically relevant regions.

**Figure 4:**
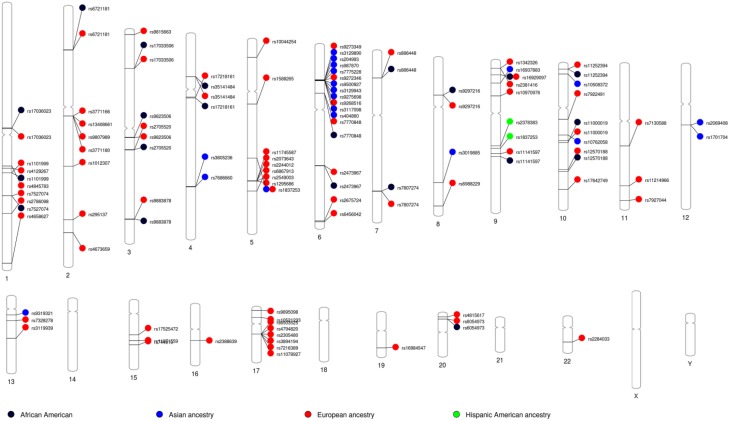
**Asthma GWAS catalog variants grouped by ancestry: Asthma related variants identified through genome-wide association studies (GWAS) NHGRI catalog of published GWAS was searched**. PhenoGram was used to plot and visualize GWA catalog association results for potentially pleiotropic SNPs among across ancestry (Wolfe et al., 2013). An Ideogram of all 22 chromosomes is plotted, along with the X and Y chromosomes. Lines are plotted on the chromosomes corresponding to the base-pair location of each asthma-related SNP, and the line connects to colored shape representing the phenotype(s) associated with that SNP.

Because most GWASs involve populations of European ancestry, we examined the allele frequency patterns of 78 GWAS SNPs associated with asthma and deposited at the GWAS Catalogue site (Welter et al., 2014). We used the 1000 Genomes Project database (http://www.1000genomes.org) and the *AncestrySNPminer* (Amirisetty et al., 2012) online tool to explore these variants among African American (ASW), African (YRI), and European American (CEU) samples. The admixed African American sample (ASW) exhibited allele frequencies that are intermediate between ancestral CEU and YRI samples, suggesting admixed populations require a different gene-mapping strategy than the relatively homogenous ancestral populations (Mersha and Abebe, 2015). We recently investigated differences in candidate gene association between asthmatic children of European ancestry and African American ancestry using the high-throughput genotyping custom Illumina Golden Gate assay (http://www.illumina.com) in combination with the Greater Cincinnati Pediatric Clinic Repository cohort (Butsch Kovacic et al., 2012). To account for population substructure and admixture, AIMs were selected on the basis of our recent work (Baye et al., 2009). The analyses unveiled two genes—*IL4* (among individuals of European ancestry) and *INSIG2* (in populations of African American ancestry)—that were associated with asthma (Baye et al., 2011b). Our data suggests that the genetic architecture of asthma in European Americans ancestry may be different from the genetic architecture of asthma in African Americans. We also observed ancestry-based allele “flip-flop” that could also attributed to differences in the genomic architecture between the two groups (Kovacic et al., 2011; Baye et al., 2011b). This observation is consistent with the longstanding observation that asthma prevalence differs by race. By using the results of recently published SNPs from the GWAS catalog (www.genome.gov/gwastudies) and PhenoGram visualization software (Wolfe et al., 2013), we demonstrated the shared and unique etiology of asthma as grouped by ancestry (Figure 4). Chromosomes 6 and 17 show many of the genomic variants related to asthma in the European ancestry. Variants such as rs1837253 (chromosome 5) and rs16929097 (chromosome 6) shared between European ancestry and Asian ancestry as well as between African and European ancestry, respectively (Figure 4). Overlapping loci that are shared between ancestries may suggest shared molecular pathways involved in asthma etiology, which should aid in the determination of the molecular mechanisms that trigger their progression across all ancestries.

### Admixture mapping vs. genome-wide association testing

**Figure 5:**
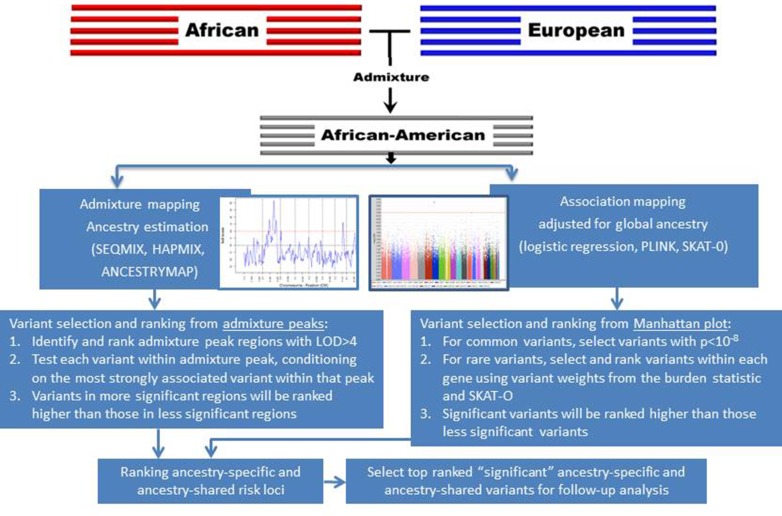
**Strategies to prioritize ancestry-specific risk loci via admixture mapping and ancestry-shared risk loci via GWAS for follow-up genotyping and analysis**.

AM uses estimates of ancestry at each SNP to test for associations with a phenotype; this is in contrast with GWASs, which compare allele frequencies with phenotype (Montana and Pritchard, 2004). GWAS is based on frequencies of SNP variants in cases vs. controls. As mentioned previously, GWASs have been implemented based on ancestral homogenety within European population, with the assumption that association is uniform across all loci (Wellcome Trust Case Control, 2007). However, for AM approach, this assumption means that any prior evidence from AM of ancestry effects is completely ignored. In admixed populations, AM has greater statistical power as compared with GWASs because, as we focus on efficient detection of genomic region of ancestral difference, we require a smaller number of genetic markers to cover the genome (Patterson et al., 2004; Smith et al., 2004). This is because, for example, in African Americans, LD extends as far as 20 cM, while LD in European ancestry rarely extends longer than 0.1 cM (Parra et al., 1998). At the same time, AM estimates ancestry of the genomic region (i.e., the region of admixture LD) and therefore does not have the resolution of a GWAS. However, AM draws our focus to a specific region of interest with 200–500 fold fewer comparisons that must be corrected using multiple comparisons techniques. Thus, the optimal settings for admixture-based approaches would be best in combination with GWASs to potentially identify ancestry-specific and ancestry-shared genetic variants in asthma (Figure 5). Results from the two largest meta-analyses of GWAS on asthma done to date, the GABRIEL and EVE consortia, indicated that some loci are ancestry specific, including *ORMDL3/GSDML* in European and Hispanic ancestry and *PYHIN1* in African-American ancestry (Torgerson et al., 2011). Recent ethnic-specific associations of rare and low-frequency variants with asthma showed association of *GRASP* and *GSDMB* variants in the Latino ancestry and *MTHFR* variants in the African ancestry samples (Igartua et al., 2015). Thus, to explain genetic basis of population differences and risks for asthma disparities, it is important to conduct genetic studies in different race, ethnicity, and admixed ancestry because genetic markers can vary from findings of European ancestry (Galanter et al., 2014; Leung et al., 2014). AM in admixed population have led to the identification of novel associations that would not otherwise have been identified in traditional GWAS.

### Joint admixture and association testing in admixed populations

Admixture and association mapping signals contain complementary information, and incorporating both signals may estimate much of the heritability missed by either of these methods alone (Manolio et al., 2009). Several methods have been developed to combine AM and association mapping (Tang et al., 2010; Lettre et al., 2011; Shriner et al., 2011; Pasaniuc et al., 2013). However, most of these methods assume independence between local ancestry and genotype. To overcome this, one can jointly test ancestry and association for a sample of admixed individuals in two steps. Step 1 involves the performance of high-density AM using local ancestry. Step 2 uses association mapping with a stratified regression approach wherein each marker's genotypes are stratified by local ancestry. Joint testing of the posterior probabilities with AM and the prior probabilities with association mapping capitalizes on the reduced testing burden of AM relative to association mapping. Thus, the combination of AM and association mapping has significant potential to effectively uncover novel variants that have been missed in previous mapping efforts and thus may be able to potentially explain much missing heritability (Manolio et al., 2009). By taking advantage of the reduced test burden of AM as compared with association mapping, Shriner et al. (2011) developed a two-step approach as follows:

$$\begin{array}{lll} f\left(y_{i}\right) & = & \beta_{0}+\beta_{1}A_{ij}+\beta_{2}\bar{A_{i\cdot }}+\epsilon_{i},and\\ f\left(y_{i}^{\left(k\right)}\right) & = & \beta_{3}^{\left(k\right)}+\beta_{4}^{\left(k\right)}G_{ij}^{\left(k\right)}+\beta_{5}^{\left(k\right)}{\bar{A_{i\cdot }}}^{\left(k\right)}+\epsilon_{i}^{\left(k\right)} \end{array}$$

where *y*_*i*_ is the observed phenotype of the *i*^*th*^ individual, *f*(·) is the link function, *A*_*ij*_ is the local ancestry for the *i*^*th*^ individual at the *j*^*th*^ marker (e.g., for African Americans, 0, 1, or 2 copies of African chromosomes, $\bar{A_{i\cdot }}$ is the global ancestry for the *i*^*th*^ individual, and ε_*i*_ is the residual error.

Here β_1_ is the coefficient for the AM effect and $\beta_{4}^{\left(k\right)}$ is the coefficient for stratum specific genotype association. Joint framework for testing the effects of ancestry via admixture and genotype via association mappings results will motivate additional analyses in existing GWAS or admixture based NGS studies, potentially detecting additional loci which might be missed otherwise.

### Rare variants and admixture mapping

The bulk of human genetic variation is the result of *common variants* that were inherited from the ancestral African population before the “out-of-Africa” migration and that are present across the world. *Rare variants* have occurred in recent human history and therefore they may not be shared among different ancestral populations (Rosand and Altshuler, 2003; Frazer et al., 2009; Baye and Wilke, 2010). The recent admixture between geographically isolated populations (e.g., Europeans, Africans, Native Americans) has had a marked effect on the genetic variation of rare variants. For rare variants, there have been significant changes in allele frequency during the time since the separation of the parental populations; these loci can be leveraged to identify the loci that affect clinically relevant traits (Chakraborty and Weiss, 1988; McKeigue et al., 2000; Shriver et al., 2003; Reich et al., 2005). Since rare variants might have arisen after populations diverged, they are more likely to be specific to certain populations and might also be overrepresented in specific ethnic groups (Chakravarti, 1999; Keen-Kim et al., 2006). A study showed the reduced sharing of rare variants as compared with common variants even among closely related populations, such as Chinese and Japanese populations as well as Northern and Southern European populations (Gibson, 2011). This lack of sharing can be explained by ancestry divergence as a result of local adaptation, and expected to increase with sample size. Thus, the presence of rare variants in only one of the ancestral populations might explain the difference in disease prevalence, including asthma. If the source of an association with a gene is a rare allele, population genetic theory suggests that, in the absence of selection, the allele will have established itself in the population more recently than most common alleles.

Although GWASs have successfully identified numerous asthma associations (Zhang et al., 2012), their reliance on “common disease/common variants” hypotheses with notoriously small effect sizes has become a barrier to further progress. For example, as of January 2015, 33 GWASs of asthma and asthma-related traits have yielded 78 common variants, which collectively account for only 5% of the variance in asthma susceptibility (Ramasamy et al., 2012). This leaves most of the high-risk mutations that contribute to asthma unidentified (“missing heritability”) (Manolio et al., 2009). In addition, growing evidence suggests that rare variants exhibit considerably larger effect sizes relative to common variants (Rivas et al., 2011; Gudmundsson et al., 2012). A recent study identified a low-frequency variant (MAF = 1.1%) for adiponectin levels that explained a large percentage (17%) of phenotypic variance in the sample (Bowden et al., 2010). Moreover, many of the variants that have been identified in GWASs are largely from individuals with European ancestry and thus are not representative of the admixed AA genome (Carlson et al., 2013). Recent studies showed that only 81% of the common SNPs from African ancestry are represented in the GWAS commercial genotyping platforms as compared with 94% of those from European ancestry (Clark et al., 2005; Consortium et al., 2007; Baye et al., 2009). In addition, recent study using exome data showed that rare variants in asthma are ancestry specific (Igartua et al., 2015). AM can be particularly useful when mapping rare risk alleles from admixed populations that have important frequency differences between ancestral populations (Mersha and Abebe, 2015). Studies have shown an accumulation of rare variants in the extreme range of the phenotype; such variants operate equally across all levels of the phenotype (Coassin et al., 2010; Gloyn and McCarthy, 2010; Johansen et al., 2010). Sequencing from the upper and lower 10% tails of the phenotype distribution increases the power of AM by increasing the frequency differences of risk variants between the two extreme phenotypes, thus requiring smaller sample sizes for the identification of novel regions and variants (Pritchard, 2001; Plomin et al., 2009; Lanktree et al., 2010; Guey et al., 2011; Li et al., 2011a,b; Barnett et al., 2013; Benitez et al., 2013). The strategy of selecting individuals from the extremes of the phenotypic distribution for maximum allele frequency difference has been successfully applied to quantitative traits such as plasma low-density lipoprotein levels (Cohen et al., 2006). Ancestry-based rare variant studies in admixed groups can play a great role in the mapping of asthma susceptibility loci, and thus have the potential to map the genetic basis of inter-ethnic differences. The joint modeling of rare and common variants signal could potentially explore independent contributions from each effect on asthma and increase our power to explain much of the missing heritability (Manolio et al., 2009; Dickson et al., 2010; Baye et al., 2011c).

### Genetic ancestry and asthma clinical outcome variables

**Figure 6:**
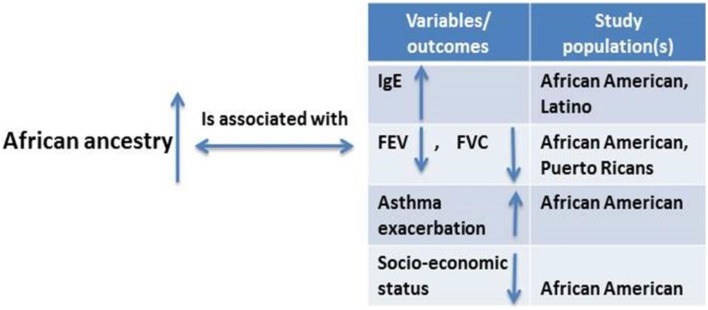
**Studies considering the relationship between degrees of African ancestry proportion and asthma and asthma-related outcomes**.

Asthma outcome variables such as forced vital capacity (a measure of lung size), forced expiratory volume in 1 s (a standard measure of lung function), and the ratio of forced expiratory volume in 1 s to forced vital capacity could be used to explain differences in disease prevalence among racial groups (Salari et al., 2005; Menezes et al., 2015). African ancestry was inversely related to FEV1, FVC, and FVC (Kumar et al., 2010) (Figure 6). A study showed that each percentage point of increase in African ancestry was associated with an 8.9 ml decrease in forced expiratory volume in 1 s and an 11.8 ml decrease in forced vital capacity (Ortega et al., 2014). A higher degree of African ancestry was associated with a greater likelihood for an asthma-related physician visit and a greater frequency of urgent or emergency department visits. Total higher serum immunoglobulin E levels and lower responsiveness to bronchodilators have also been observed among individuals with higher African ancestry with persistent asthma as compared with whites and other racial groups (Kumar et al., 2010). All evidence demonstrated that measures of genetic ancestry—rather than race/ethnicity classification—could improve clinical care for people of mixed race. Adding measured ancestry to lung function prediction equations in asthma severity analysis reduced misclassification rate by 5% (Kumar et al., 2010).

### Genetic ancestry and environmental risk factors in asthma

**Figure 7:**
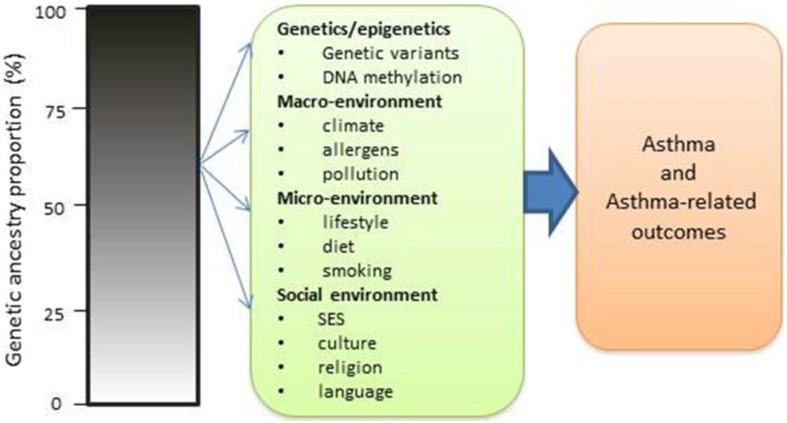
**Impact of ancestry with both genetic and non-genetic risk factors in causing asthma and asthma-related risks**.

Although genetic influences are important determinants of asthma risk, there is also compelling evidence for the socioenvironmental contributions. However, there is lack of studies on the role that socioenvironmental factors play on asthma risk. Gravlee et al. (2009) and many others (Kaufman et al., 1997; Non et al., 2010, 2012) have indicated that ancestry may serve as a means of tracking environmental influences such as socioeconomic status and sociocultural factors that may contribute to asthma disparity (Figure 7). Thus, individuals with mixed ancestry provide an effective way to disentangle the effects of ancestry from those of the environment. For example, if a greater African ancestry is observed across the genome in asthmatic patients relative to controls without significant rise in local ancestry at a particular locus, this may point to a stronger role for non-genetic factors (e.g., exposures to traffic, mold, cigarette smoke, socioeconomic status) in asthma risk (Deo et al., 2007; Gravlee et al., 2009; Winkler et al., 2010). However, the absence of significant chromosomal increase does not exclude multiple genes with small effects that contribute to asthma in persons of African ancestry. It does suggest that there are no large-effect genes that explain asthma risk. The availability of dense markers, environmental and social variables, and new analytical tools allow for a more rigorous approach in which the effects of ancestry and environmental determinants can be disentangled and comprehensively investigated in asthma.

In recent years, the increase in asthma prevalence worldwide has been especially noticeable in the developed world, where asthma prevalence has increased from 3.6% in 1980 to 10% in 2007 (CDC, 2011; Norman et al., 2013). The rise in asthma prevalence is occurring at a faster rate than changes in allele frequencies of the genome should allow, and this may indicate the role of underlying environmental risk factors in the modulation of asthma (Huang et al., 2015). Such observations are consistent with the highly publicized analogy, “*genetics loads the gun, but the environment pulls the trigger*”(Olden and White, 2005). That is, one can inherit the genetic predisposition to develop a disease but will do so only if or when exposed to the environmental trigger. Today, it is believed that a large component of asthma variance responds not only to the individual contributions of predisposing genes but also to the extent to which such genes interact with the environment (Baye et al., 2011a). Thus, many factors in addition to ancestry can influence asthma severity, and the study of genetic ancestry with environmental exposure and other social determinants of health are critical to understand the etiology of asthma.

In a study of the Wayne County Health Environment Allergy and Asthma Longitudinal Study (WHEALS) cohort, 601 WHEALS mothers who identified themselves as white (*n* = 216) or African American (*n* = 385) were studied. Self-identified African American women were more likely to be sensitized to at least one allergen (allergen-specific immunoglobulin E, ≥0.35 IU/mL) as compared with white women [odds ration (OR) = 2.19]. However, genetic ancestry based on AIMs was not significantly associated with allergic sensitization (OR = 1.34) after adjusting for location of residence (urban vs. suburban). Similarly, Keet et al. (2015) showed that “black race, Puerto Rican ethnicity, and lower household income” to be strong independent risk factors for asthma exacerbation and ED visits for asthma. These data suggest that genetics may not explain all of the observed racial disparity associated with allergic sensitization (Yang et al., 2008). Among children who were approximately 2 years old, the Boston Birth Cohort genotyped 150 AIMs and found that African ancestry was associated with food sensitization (OR = 1.07 for a 10% increase in African ancestry). However, there was no adjustment for location of residence, income, maternal education, or other socioeconomic factors. The understanding of how genetic ancestry interacts with socioenvironmental risk factors to impact asthma-related traits is an area that requires further exploration (Mersha and Abebe, 2015). However, investigating these interactions will require the following: (1) the a priori identification of which environmental parameters will be considered relevant for the analysis; (2) methodologies to allow for the simultaneous consideration of multiple genes and perhaps multiple environmental variables and various ancestry proportions; and (3) an effort to overcome the perceived assumption of genetic and environmental homogeneity when evaluating admixed populations.

### Asthma genetic heterogeneity and pharmacogenomic intervention

Race variations in response to medication were observed as early as the 1920s (Kalow, 2004). Wu et al. (2005) summarized many clinical trials results for antihypertensive medications and found that some of these drugs were more effective for African Americans than for those with European ancestry and vice versa. Lin et al. (2005) provided an example of some typical population complexities seen in the application of personalized medicine. Differences in drug outcome measure by race suggest that drug metabolism in African Americans may be governed by different genetic factors than European Americans, and may lead to different options for risk identification and intervention. This potential genetic difference in drug outcome is supported by recent work identifying associations between individuals' proportion of African ancestry and drug related phenotypes (Ortega and Meyers, 2014). By analyzing the drugs in ancestry dependent manner, we will increase power and able to determine whether ancestral variation is associated with the class of drugs or whether the ancestral variation affects the drugs differently (that is, interaction). However, the majority of asthma clinical studies have been conducted with the use of individuals of European ancestry, and their results have been generalized to all patients, irrespective of their racial or ethnic groups (Deo et al., 2009). For example, the frequency of alleles related to asthma medication drugs, such as the β2-adrenergic receptor gene ADRB2, differs between African American, Caucasian, and Chinese populations (Sayers and Hall, 2005). There exist a significant pharmacogenetic difference in β2-adrenergic receptor polymorphisms and bronchodilator responses to albuterol even between the two largest Latino groups: Mexicans and Puerto Ricans (Suarez-Kurtz, 2005). There is also a momentous debate about the “race-targeted” drug BiDil, which is used for treating heart failure in African Americans (Brody and Hunt, 2006). Recent research in the field of pharmacogenomics has used DNA information to improve the prediction of drug response. However, many models incorporate race but do not consider ancestry information when assessing host–drug interactions, thus limiting its predictive ability. Warfarin dosing algorithms that are developed for well-defined racial groups are not applicable to the heterogeneous admixed population because admixed populations deviate from the idea of race- or ethnicity-based classification. Given that the variation in the response to therapy could be largely due to genetic differences, the field of asthma is well suited to pharmacogenetic investigations to develop and provide a genetic basis for “individualized therapy” (Lin et al., 2005).

### Admixture based population substructure and socioenvironmental factors in asthma studies

Hidden population substructure in human populations has become a major issue for studying complex diseases including asthma, especially in admixed populations (Bryc et al., 2015; Mersha and Abebe, 2015). Population stratification arises when ancestry is associated with a phenotype of interest. Understanding the consequences of admixture based population substructure in admixed populations is important, because admixture can be both a confounding factor and a source of statistical power to map asthma-related genes (Redden et al., 2006). Population stratification is the existence of groups of individuals within a population that arise as a result of reproductive isolation from the rest of the population. For example, several association studies (candidate gene studies or GWASs) have case-control study designs in which the frequency of an allele or genotype at a locus in the study patients is compared with its frequency in an unaffected control population. This study design is subject to population stratification as a result of genetic admixture, which occurs when the cases and controls are unintentionally drawn from two or more racial or ethnic groups or subgroups in disproportionate frequencies (Mersha and Abebe, 2015). If one of these subgroups has higher disease prevalence than the others, stratification occurs, because that subgroup can be overrepresented in the cases and underrepresented in the controls. For the bias caused by population structure to exist, both of the following must be true: (1) the frequency of the variant of interest varies significantly by race; and (2) the background disease prevalence varies significantly by race (Wacholder et al., 2000). In addition, environmental exposures that differ between ancestry groups may confound or interact with genetic factors. It can be difficult to fully account for confounding with socioeconomic status including income because disparities in wealth, educational opportunities, family structure, and employment by race or ethnicity are even higher than what is represented by income (Sampson et al., 2008). The standardization of environmental exposure assessment methods is needed, and statistical analyses have to be carefully adjusted for demographic and environmental factors (Thomas et al., 2012). African Americans and Latino Americans often have lower socioeconomic statuses, and these are often associated with environmental factors such as diet, the presence of allergens, and pollution exposure. These factors can have a direct effect on the development of asthma and need to be carefully adjusted for during statistical analysis.

To date, several algorithms have been developed to adjust for population stratification including genomic control, structured association, and principle component analysis (Devlin and Roeder, 1999; Pritchard et al., 2000b; Price et al., 2006). Detail comparison of these methods are provided by Zhang et al. (2008a). The common practice to correct population substructure involves estimating global genetic ancestry for each sample (Reich et al., 2008; Torres-Sanchez et al., 2013). This correction is often accomplished by genotyping a set of AIMs (or high density markers) and then using the known reference populations for evaluation with the use of either principal components analysis (for a continuous estimate of ancestry group) (Price et al., 2006) or cluster analysis (for a categorical ancestry assignment) (Pritchard et al., 2000b). Global ancestry measures are used to stratify individuals or to include them as covariates for adjustment in statistical analysis. In using AIMs or high density markers, the population under study should have the same substructure as the population in which the AIMs or high density markers were discovered. In admixed populations when the disease prevalence differs between ancestries (e.g., populations with asthma), global ancestry is a confounder that must be adjusted for to obtain valid inference.

### The application of admixture mapping in the context of next-generation sequencing

As computational power increase and cost of sequencing has decreases, next-generation sequencing (NGS) strategies are now being used to find causal variants associated with asthma (Dewan et al., 2012). With the availability of high-density NGS data, the ancestral origin of chromosomal segments can be inferred with high accuracy. The estimation of ancestral proportion segments has practical implications for both accounting population structure in association testing and AM. Failure to adequately account for ancestry variation may lead to spurious results in large-scale association studies (Baye et al., 2011c). With increasing knowledge gained through sequencing, high-density genotyping arrays of diverse populations, and the construction of a high-resolution map of admixture, a recent study showed that populations that were assumed to be homogeneous (e.g., European Americans) are in fact admixed and that AM could be feasible for the mapping of ancestry-associated variants in this population (Bryc et al., 2015). Admixture may work well for sequencing data, because it relies on consistent genetic differences between populations that may exist at the rare variant level. In contrast with common variants, rare variants are not shared across divergent populations, because they have either arisen relatively recently or their frequencies have been influenced by population history (e.g., the out-of-Africa expansion, natural selection). It has been shown that haplotype-based approaches allow for the finer reconstruction of genetic structure as compared with single-marker genotypes (Conrad et al., 2006; Lawson et al., 2012). The challenge with the NGS approach is that the frequency rare variants are unknown, and data quality will be an issue. For rare variants-based NGS admixture mapping, we suggest a haplotype-based ancestry identification approach to determine the contributions of different ancestral populations. This approach will provide multiple rare variants in a given haplotype will provide power to identify previously unreported contributions from African, European, and Native American populations in the ancestry of American admixed populations (Montinaro et al., 2015). Although NGS data can be used to identify genetic variants (e.g., copy-number variants, insertions, deletions, other structural variants, regulatory variants) and DNA methylation sites, new methods are required to employ AM for these data.

## Limitations of admixture mapping

The underlying assumption in AM is that the risk allele occurs at different frequencies among ancestral populations, i.e., affected individuals share an excess ancestry from the ancestral population with the highest frequency of the risk allele. Thus, the power of AM relies strongly on the allele frequency difference of the causative variants. AM has no power if the frequency of asthma-associated variant is similar between the ancestral populations. Another limitation of AM is that, if a region in the ancestral genome undergoes selection for another cause (e.g., an infectious disease) that is unrelated to asthma risk *per se*, this could lead to spurious results. Most alleles that vary across geographic regions may also have developed in relation to environmental exposures (e.g., alleles related to malaria resistance). As a result, a shared or unique allele might reflect similar or different environmental exposures rather than shared or unique ancestries (Bolnick et al., 2007; Frudakis, 2008). Ultimately, the best means of protection from false-positive errors (e.g., the identification of loci unrelated to disease) is replication in an independent study population. Most AM methods make use of reference ancestry panels that serve as proxies for the ancestral populations in the admixture. Good reference panels may not exist for many populations, including the Native American ancestral component that is present in many Hispanic populations.

Several methodological challenges also exist in admixed populations, including the following: (1) The history of human admixture is not under experimental control or even necessarily known. The number of generations is usually estimated. (2) Ancestral populations are not available for study. Ancestry-specific allele frequency is re-estimated within the admixed population, with prior estimates based on sampling unadmixed modern descendants. However, the true number of ancestral populations in an admixed population is not known. (3) The exact mix of ancestral populations that contributes to the admixed gene pool cannot be sampled. (4) Human racial groups are not inbred strains, and markers with 100% frequency differentials are rare. Thus, we cannot unequivocally infer ancestry at a specific locus from a marker genotype. (5) Despite recent interest in applying admixture methods to diverse populations (Beaumont et al., 2001; Wen et al., 2004; Reich and Patterson, 2005; Semon et al., 2005; Winkler et al., 2010), most methods were developed with the use of an isolation (or intermixture) model (which assumes that admixture occurs only at the first generation and is then followed by random mating within the admixture population) and tested via the simulation of randomly generated genotypes and allele frequencies (from uniform distribution) for individuals in an admixed population (Long, 1991). However, human admixing is continuous over time and it is unlikely this model accurately represents the complexity of the process by which admixed human populations were formed (Waples and Gaggiotti, 2006). These admixture models create different LD patterns (Long, 1991). (6) There are currently few software options which offer admixture analysis of dense marker data from more than two admixed populations. In addition, currently available software estimate admixture but not ancestry (Vaughan et al., 2009). *Admixture* is an error containing proxy measurement of *ancestry*, i.e., Admixture_i_ = Ancestry_i_ + Error_i_. The error could be due to measurement (incomplete coverage of genome), missing data (imprecise allele frequencies for founding populations), biological variation (meiosis/recombination) and other errors such as genotyping, information content of markers (Divers et al., 2007). (7) Ancestral population LD tends to confound with admixture LD, and methods are required to unravel admixture LD from ancestry LD.

To evaluate some of these limitations through simulation models and testing randomly generated ancestral and admixed human populations, it may be relevant to use 1000 Genomes Project datasets as a starting point in the simulation process and to follow the gradual admixture model, which assumes that admixture occurs at each generation (Ewens and Spielman, 1995). In addition, Vaughan and colleagues (Vaughan et al., 2009) presented the relevance of plasmode datasets for comparing the true and estimated admixture values using root mean square error (RMSE) accuracy measures (Tang et al., 2005). Final limitations include samples of ancestry reference sets that are comprised of the genomes of relatively few sampled individuals who are themselves from a relatively small number of ancestral samples from geographically restricted regions. To what extent this panel represents the current status of admixed populations is debatable. Most methods estimate ancestry at a continental scale, thereby making the identification of multiple sources from the same continent or region challenging. Regional genetic differentiation and differing patterns of shared ancestry within regions may provide clear signals of historical demographic events. Ultimately, AM, as hypothesis-generating tool, can only coarsely localize loci that contribute to asthma; it cannot fine-map important variants (Reich et al., 2007). Follow-up “zoom in” analysis will be required to identify the specific variants associated with asthma. Therefore, it is prudent to recognize the limitations of ancestry reference samples, markers, and current methodological challenges in genetic and genomic studies of admixed populations.

## Summary and conclusion

Admixed populations arise when two or more previously isolated populations start interbreeding. As DNA recombination breaks and rejoins to form new ones, genomic mosaicism with different genetic ancestry segments are created and further reshaped and rearranged by recombination in each generation. As the number of generations increases, the ancestral chromosomal segments from different parental populations are spliced into shorter pieces. These segments of DNA (blocks of haplotypes) have distinguishable ancestral origins and provides valuable information for AM. Studies have shown that the frequency of alleles associated with asthma differ by race. Differences among individuals of African and European descent, for example, and the admixture nature of the African American population create an ideal opportunity of AM to map ancestry-specific variants. Ancestry-based gene-mapping approaches in admixed groups can be expected to play a great role in the mapping disease susceptibility loci, and to elucidate the genetic basis of inter-racial differences. Furthermore, they will provide valuable opportunities to study the interactions of race, genetics, culture, and environments.

Most genomic studies in admixed populations use commercial genotyping arrays that are developed based on reference panel from relatively homogeneous European ancestry population, and may not adequately tag relevant variations in admixed populations. Increasing availability of polymorphic molecular markers from directly sequencing multiple individuals/populations using NGS (e.g., the 1000 Genomes Project), allows admixture proportions to be accurately estimated at unprecedented local detail. Consequently, researchers are able to distinguish between closely related individuals of variable with varying ancestry and to determine the ancestry of genomic segments at a fine scale across an individual's genome. Although recent technologies make it possible to sequence and generate millions of high-density variants to identify ancestry-specific markers, well-characterized and standardized phenotyping still lags behind.

Until now, AM approaches for asthma have focused on single-locus ancestry effects, whereas comprehensive analyses of haplotype–ancestry blocks from multiple loci or ancestry–environment interactive effects in the context of disease genetics have not yet been incorporated. Studies focusing on accurate and stringent phenotyping and genotyping at multilocus ancestry markers—including other types of variants (e.g., copy-number variants, insertions, deletions, other structural variants, regulatory variants) and omics data (e.g., DNA methylation, metabolome)—and that take into account potential population stratification due to intrinsic and extrinsic environmental stimuli will facilitate ancestry-based asthma gene mapping. Taken together, investigating the genetic architecture of asthma in diverse ancestral and admixed populations will help to understand its etiology and perhaps shed light on the disparities seen in childhood asthma risk between races.

## Author contributions

TM conceived and wrote the manuscript.

### Conflict of interest statement

The author declares that the research was conducted in the absence of any commercial or financial relationships that could be construed as a potential conflict of interest.
